# Human T cell recognition of the blood stage antigen *Plasmodium *hypoxanthine guanine xanthine phosphoribosyl transferase (HGXPRT) in acute malaria

**DOI:** 10.1186/1475-2875-8-122

**Published:** 2009-06-07

**Authors:** Tonia Woodberry, Alberto Pinzon-Charry, Kim A Piera, Yawalak Panpisutchai, Christian R Engwerda, Denise L Doolan, Ervi Salwati, Enny Kenangalem, Emiliana Tjitra, Ric N Price, Michael F Good, Nicholas M Anstey

**Affiliations:** 1 International Health Division, Menzies School of Health Research, Charles Darwin University, Darwin, Australia; 2 The Queensland Institute of Medical Research, Brisbane, Australia; 3 National Institute of Health Research and Development, Ministry of Health, Jakarta, Indonesia; 4 National Institute of Health Research and Development-Menzies School of Health Research Research Program and District Health Authority, Timika, Papua, Indonesia; 5 Centre for Vaccinology & Tropical Medicine, Nuffield Department of Clinical Medicine, Churchill Hospital, Oxford, UK

## Abstract

**Background:**

The *Plasmodium *purine salvage enzyme, hypoxanthine guanine xanthine phosphoribosyl transferase (HGXPRT) can protect mice against *Plasmodium yoelii *pRBC challenge in a T cell-dependent manner and has, therefore, been proposed as a novel vaccine candidate. It is not known whether natural exposure to *Plasmodium falciparum *stimulates HGXPRT T cell reactivity in humans.

**Methods:**

PBMC and plasma collected from malaria-exposed Indonesians during infection and 7–28 days after anti-malarial therapy, were assessed for HGXPRT recognition using CFSE proliferation, IFNγ ELISPOT assay and ELISA.

**Results:**

HGXPRT-specific T cell proliferation was found in 44% of patients during acute infection; in 80% of responders both CD4^+ ^and CD8^+ ^T cell subsets proliferated. Antigen-specific T cell proliferation was largely lost within 28 days of parasite clearance. HGXPRT-specific IFN-γ production was more frequent 28 days after treatment than during acute infection. HGXPRT-specific plasma IgG was undetectable even in individuals exposed to malaria for at least two years.

**Conclusion:**

The prevalence of acute proliferative and convalescent IFNγ responses to HGXPRT demonstrates cellular immunogenicity in humans. Further studies to determine minimal HGXPRT epitopes, the specificity of responses for Plasmodia and associations with protection are required. Frequent and robust T cell proliferation, high sequence conservation among *Plasmodium *species and absent IgG responses distinguish HGXPRT from other malaria antigens.

## Background

Malaria remains a major public health problem and approximately 1 million people continue to die annually from *Plasmodium falciparum *malaria [1]. Correlates of immune protection remain poorly characterized. Of the current malaria vaccine strategies, few have been shown to protect humans from malaria. Irradiated sporozoites confer protection [2,3] and ultra low dose blood parasitized red blood cells (pRBC) have been shown to induce potent cell mediated immunity that may contribute to enhanced resistance to *P. falciparum *infection [4,5]. The pre-erythrocytic stage vaccine RTS,S confers partial but not complete protection against clinical disease [6-8], and a DNA-MVA heterologous prime-boost regimen can protect against sporozoite challenge [9]. All of these strategies elicit cellular immune responses, which contribute to protection [10,11]. Since T cell mediated protection has been demonstrated in the absence of antibodies [4,12-17], the identification of parasite antigens targeted by cellular responses is required to better understand the development of immunity to disease and to identify novel antigens that warrant consideration as potential vaccine candidates.

In mice, the *Plasmodium *purine salvage enzyme, hypoxanthine guanine xanthine phosphoribosyl transferase (HGXPRT), is a target of protective T cells as evidenced by adoptive transfer studies [18]. Because of this, HGXPRT has been proposed as a novel vaccine candidate. HGXPRT is located in electron-dense regions within merozoites and in vesicles within the cytoplasm of infected red cells [19]. Since *P. falciparum *is incapable of *de novo *purine synthesis, HGXPRT is an important enzyme, and is highly conserved amongst *Plasmodium spp. *[20]. The key role of HGXPRT, the substantial sequence homology and the demonstration that T cells specific for *Plasmodium yoelii *and *P. falciparum *HGXPRT in the absence of antibodies confer protection against pRBC challenge in a mouse model raises the question as to whether this region is recognized by humans. Accordingly, this study was designed to determine whether T cell responses to *Plasmodium *HGXPRT, a blood stage antigen, are induced in humans following natural *Plasmodium *exposure. These data confirm that HGXPRT is a target of cell-mediated immunity in humans with frequent and robust T cell responses detected during acute infection.

## Methods

### Study subjects and samples

Subjects were recruited in Timika, a lowland region of Papua, Indonesia, with endemic unstable malaria transmission of multidrug-resistant *P. falciparum *and *Plasmodium vivax *and annual malaria incidence of 876 per 1,000 person-years [21-23]. Venous blood was collected from patients with acute uncomplicated falciparum malaria who presented to community or hospital outpatient clinics with fever or history of fever within 48 hours and any parasitaemia, the majority of whom were enrolled in trials of artemisinin combination therapy [22,23]. In a subset of these patients longitudinal samples were collected approximately 7 and 28 days following anti-malarial drug treatment. Two groups of controls were enrolled; (i) asymptomatic malaria-exposed controls, resident in Timika district for at least two years, with no fever or symptoms of malaria within the preceding two weeks and (ii) healthy Australian Red Cross Blood Service donors and laboratory volunteers not exposed to malaria. Plasma and PBMC were cryopreserved for later analysis.

Written informed consent was obtained from all subjects. The study was approved by the Ethics Committees of the National Institute of Health Research and Development, Ministry of Health, Jakarta, Indonesia, Menzies School of Health Research and the Australian Red Cross Blood Service.

### Recombinant protein, synthetic peptides and mitogens

*Plasmodium falciparum *cDNA K1 isolate, PlasmoDB PF10_0121 [24] coding for HGXPRT was cloned into a pT7-7 expression vector and subsequently transformed into SΦ606 (*ara*, Δ*pro-gpt-lac, thi, hpt, F*^-^) *E. coli *cells. The enzyme was then purified to homogeneity to a concentration of ≈7.5 mg ml^-1 ^as described [25]. Mass spectrometry confirmed a molecular weight of 26,231 Da. Recombinant protein was tested for toxicity and mitogenicity in bulk splenocyte cultures prior to use. 1.6–2.0 μg of HGXPRT protein was used in functional assays. Additionally, twenty two peptides corresponding to the entire *P. falciparum *K1 isolate HGXPRT sequence [26] were produced at the Queensland Institute of Medical Research. Peptides were 20 amino acids in length, overlapping each other by 10 amino acids. 1.6 μg of individual peptides at a purity of >85% were evaluated in functional assays.

Recombinant hexahistidine tagged full-length *P. falciparum *merozoite surface protein 5 (MSP5) [27] and the mitogen phytohaemagglutinin (PHA, Sigma, Missouri, USA) at 5 μg/ml, were used as positive controls.

### Sample evaluation

PBMC from 73 patients with acute and/or convalescent malaria were tested for HGXPRT protein recognition in proliferation and/or ELISPOT assays. The limited number of available cells restricted the number of patients in whom both assays could be performed (Table 1 and Table 2) to 12 subjects Plasma from all 73 subjects evaluated for cellular responsiveness were tested for HGXPRT-reactive immunoglobulins. PBMC from 15 healthy Australian blood donors not exposed to malaria were tested as controls in the proliferation and ELISPOT assays. Plasma from 37 healthy Australian blood donors were tested as controls in the HGXPRT ELISA.

**Table 1:** Patients with acute and convalescent malaria in which proliferative responses were tested

	***Day 0***		Day 28*
	HGXPRT proliferation	No HGXPRT proliferation	
Subjects	15	19	12
Mean age (range)	24 (7–55)	25 (12–44)	25 (8–43)
Female/male	6/9	7/12	5/7
Mean parasites/μL (range)	13 153 (2 324–30 800)	24 102 (255–305 200)	0

* responses were not stratified by HGXPRT proliferation due to the paucity of proliferation at day 28.

**Table 2:** Patients with acute and convalescent malaria in which ELISPOT responses were tested

	Day 0*	***Day 28***	
		HGXPRT response	No HGXPRT response
Subjects	12	10	9
Mean age (range)	25 (3–50)	23 (13–43)	30 (7–60)
Female/male	2/10	4/6	4/5
Mean parasites/μL (range)	4911 (423–15 912)	0	0

* responses were not stratified by HGXPRT recognition due to the paucity of IFNγ secretion at day 0.

### CFSE proliferation assay

PBMC resuspended at 1 × 10^6^/mL in PBS 0.1% FCS (GibcoBRL, Life Technologies) were stained with 0.4 μM CFSE (Molecular Probes, CellTrace, Oregon, USA) for five minutes at 37°C and washed according to the manufacturer's instructions. PBMC were then resuspended in RPMI-1640 medium supplemented with 10% FCS (GibcoBRL), 2 mM glutamine, 100 μg per ml streptomycin and 100 units per ml penicillin (GibcoBRL), and incubated at 37°C in 5% CO_2 _for six days in the presence or absence of antigen or peptides. On day 6 cells were stained with cell surface antibodies (anti-CD3, CD4 and CD8; Pharmingen, BD Biosciences, CA, USA), resuspended in 1% paraformaldehyde (Sigma) and tested for fluorescence using a Becton Dickinson FACSCalibur with CellQuest™ Pro version 5.2.1. FACS data were analysed using FlowJo (version 7, Tree Star, Inc. Oregon, USA). Background proliferation was determined by measuring proliferation in media alone. Antigen specific responses were corrected for background proliferation (mean acute background being 11% [n = 37]) and responses ≥ 10% above background were considered positive) and reported as the percentage of dividing cells. Three acute and one day 28 PBMC sample were excluded from analyses because background media proliferation exceeded 20% and prohibited the determination of positive or negative responses.

### CD4^+ ^T cell depletion

A Dynal^® ^CD4 positive isolation kit (Dynal Biotech, Norway) was used in accordance with the manufacturer's instructions. Cells were stained with anti-CD3, CD4 and CD8 antibodies (Pharmingen) and analysed by flow cytometry to ensure the efficiency of CD4^+ ^T cell removal was ≥ 97%. CD4^+ ^T cell depleted PBMC were tested in proliferation assays as described above.

### Ex-vivo interferon gamma (IFN-γ) ELISPOT assay

400 000 PBMC were added to individual wells of mixed acetate plates (MAIPS4510, Millipore, UK) previously coated with 5 μg/ml anti-human IFN-γ mAb (clone 1-D1K, Mabtech, Sweden). The ELISPOT plates were incubated overnight at 37°C in 5% CO_2_, and then washed and developed with 1 μg/ml biotinylated anti-human IFN-γ mAb (clone 7-B6-1, Mabtech) followed by streptavidin-alkaline phosphatase (AP) (1:1000 Mabtech) and colorimetric AP Kit (BioRad, Hercules, CA, USA). Spots were counted by eye. Positive cytokine responses were based on a chi-square comparison of the odds ratio of IFN-γ secreting cells in the test well and control well [28]. The mitogen PHA was used as a positive control.

### HGXPRT ELISA

NUNC Maxisorp plates coated at 4°C overnight with 0.5 μg/ml HGXPRT protein were blocked for one hour with 5% skim milk in PBS containing 0.05% Tween (Sigma) (PBS-T) and washed with PBS-T. 50 μl of plasma, diluted in PBS-T (1:800 dilution), was added to the plate and the assay was incubated for one hour. Anti-human total IgG HRP (1:2000 dilution, Zymed, California, USA) was added and the colour developed using TMB (Zymed). The colour reaction was stopped with 1 M HCl and the absorbance read at 450 nm. The binding of antibodies in plasma from 37 unexposed donors was used to define the cut-off (mean OD + 3 SD) of positive responses at 38 μg/mL. ChromPure human IgG (Jackson ImmunoResearch Laboratories, Pennsylvania, USA) was used as a standard following dilution in PBS to 0–300 ng/mL, permitting quantitation of antibody responses.

### Cytometric bead array

Cell culture supernatant from CFSE labelled PBMC were collected 18, 36 and 65 hours following no antigen, HGXPRT or PHA stimulation. Supernatant samples were tested for IL-2, IL-4, IL-6, IL-10, TNF and IFN-γ using a Th1/Th2 cytometric bead array kit (BD Biosciences, California, USA) according to the manufacturer's instructions.

### Statistical methods

Data were analysed using SPSS for Windows (version 15 SPSS Inc, Chicago, Illinois, USA). The Mann-Whitney U test or Kruskal-Wallis method were used for nonparametric comparisons, and Student's t-test or one-way analysis of variance for parametric comparisons. For categorical variables, percentages and corresponding 95% confidence intervals (95% CI) were calculated using Wilson's method. Proportions were examined using χ^2 ^with Yates' correction or by Fisher's exact test.

## Results

### HGXPRT-specific T cell proliferation during acute malaria and in convalescence

**Figure 1:**
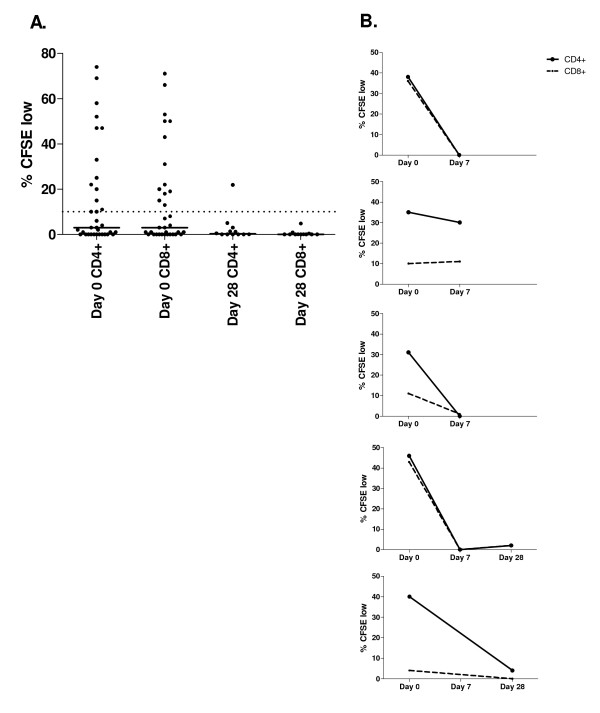
**Lymphocyte proliferation to HGXPRT in acute and convalescent malaria**. CD4^+ ^and CD8^+ ^T cell division following PBMC stimulation with HGXPRT expressed as the percentage of CFSE dim cells following background subtraction. A. PBMC response in 34 acute (day 0) and 12 convalescent (day 28) subjects. The proportion with proliferation at day 28 was significantly less than at day 0 (p = 0.035). No HGXPRT proliferation was detected in 15 malaria unexposed controls. The horizontal solid line represents the group median and the dotted line the background cut-off for positive responses. B. Longitudinal HGXPRT responses in 5 subjects during acute malaria (day 0) and 7 and 28 days after drug treatment.

To evaluate whether *P. falciparum *exposure induced T cell responses that recognize HGXPRT, cross-sectional PBMC collected during acute symptomatic malaria and one month after anti-malarial treatment were tested for proliferation in response to recombinant HGXPRT. During acute malaria HGXPRT was recognized by 44% (15/34) of subjects (Table 1). In 80% (12/15) of responders, both CD4^+ ^and CD8^+ ^T cells proliferated to HGXPRT protein with no significant differences between the two cell types (Figure 1A). In two additional subjects only CD4^+ ^T cells responded and in another only CD8^+ ^T cells proliferated. Median proliferative responses were 29% (range 10–74) and 31% (range 13–71) for CD4^+ ^and CD8^+ ^T cells respectively. There was no significant difference in the age, gender, or baseline parasitaemia between the 15 subjects with proliferative responses and the 19 without (Table 1). In the PBMC collected 28 days following anti-malarial treatment, proliferative responses were significantly less frequent, with HGXPRT CD4^+ ^T cell proliferation detected in only one subject (1/12, p = 0.035, Figure 1A).

To confirm the short duration of HGXPRT proliferative responses, eight additional acute malaria subjects with paired day 0 and day 7 and/or day 28 post treatment samples were tested for proliferation in response to recombinant HGXPRT. PBMC proliferated to HGXPRT in five subjects (62.5%) during acute malaria (day 0), in only one patient seven days after treatment and in none by day 28, despite PHA responsiveness at each time point (Figure 1B). These data confirm the cross sectional results where proliferative responses were most frequent during acute disease.

None of the PBMC from 15 malaria-unexposed controls proliferated in response to HGXPRT protein. PHA induced CD4^+ ^and CD8^+ ^T cell proliferation in all subjects tested with no significant difference in the magnitude of PHA responses between the patients with malaria and non-exposed controls.

### CD8^+ ^T cells do not proliferate after CD4^+ ^T cell depletion

**Figure 2:**
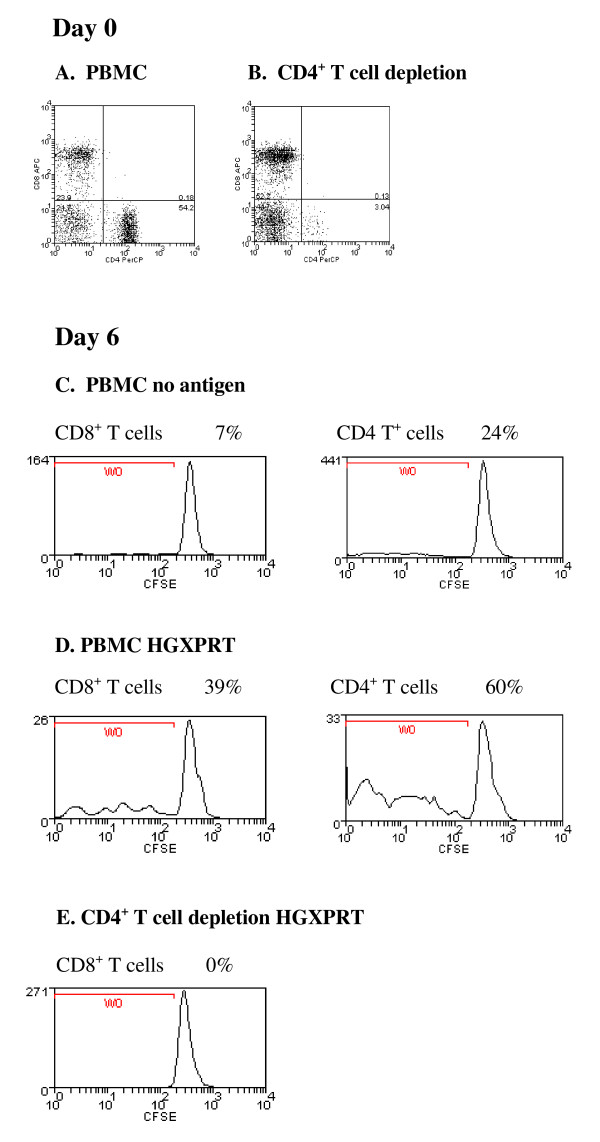
**Loss of proliferation to HGXPRT following CD4^+ ^T cell depletion**. Detection of HGXPRT-specific CD4^+ ^or CD8^+ ^T cells from a representative patient with acute falciparum malaria; (A) before and (B) after CD4^+ ^T cell depletion. Day 6 CFSE CD8^+ ^and CD4^+ ^T cell proliferative responses in response to (C) no antigen and (D) HGXPRT before and (E) after CD4^+ ^T cell depletion. The percentages represent the proportion of CFSE low CD4^+ ^and CD8^+ ^T cells in culture. A similar result was observed in two other subjects.

The frequent detection of CD8^+ ^T cell proliferation in response to soluble HGXPRT in acute malaria indicated that responsiveness differed between humans and the experimental murine malaria model [18]. To determine whether the HGXPRT CD8^+ ^T cell proliferation was dependent on CD4^+ ^T cell co-activation, CFSE proliferation was tested following CD4^+ ^T cell depletion in three acute malaria subjects who had demonstrated CD4^+ ^and CD8^+ ^T cell responses to HGXPRT. In each subject, CD8^+ ^T cells failed to proliferate in response to HGXPRT following CD4^+ ^T cell depletion (Figure 2) despite responding to PHA. These data suggest a general requirement for CD4^+ ^T cells for optimal CD8^+ ^T cell proliferation in response to HGXPRT protein in the *in vitro *cell culture system. Non-depleted PBMC from three acute symptomatic exposed and three unexposed subjects that did not proliferate in response to HGXPRT also failed to respond following CD4^+ ^T cell removal. This suggests that suppression by regulatory CD4^+ ^T cells, known to be increased in falciparum malaria [29], did not account for the failure to respond to HGXPRT.

### Recognition of HGXPRT peptides

**Figure 3:**
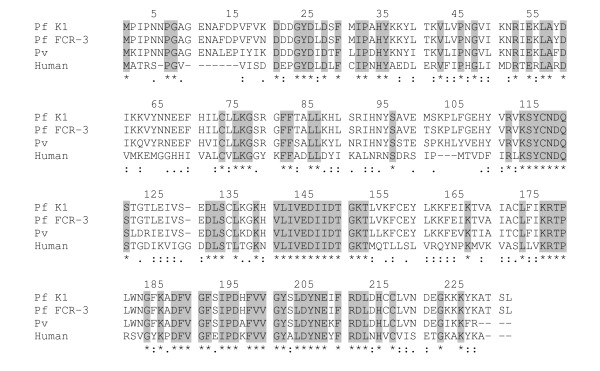
***Plasmodium *and *Homo sapiens *HG(X)PRT sequence alignment**. Sections highlighted in grey, and marked with an asterix [*] show identical amino acids in the four sequences. A colon [:] indicates highly conserved amino acids and a single dot [.] indicates reasonable conservation between the Plasmodial (accession number; XP_001614435, P07833, P20035) and human HG(X)PRT (NP_000185) sequences.

The frequent detection of HGXPRT T cell proliferation in PBMC from people with acute malaria suggests that HGXPRT is antigenic. To partially map the region within the protein targeted by T cell responses, peptides 20 amino acids in length with a 10 amino acid overlap based on the *Plasmodium *K1 sequence were synthesized. The alignment of *P. falciparum*, *P. vivax *and human HG(X)PRT protein sequences shows 39% identity (Figure 3). Due to constraints on PBMC availability from acute malaria patients, we combined peptides to create an N-terminal pool (containing 10 peptides covering the first 110 amino acids) and a C-terminal pool. Both peptide pools were tested for the ability to stimulate T cells from eleven acute malaria subjects in CFSE proliferation assays.

T cells from 36% (4/11) of subjects proliferated to the peptide pools. The N-terminal peptide pool was recognized by two subjects (with CD4^+ ^T cell proliferation in one subject and CD4^+ ^and CD8^+ ^T cells proliferation in the other) and the C-terminal peptide pool was recognized by two subjects (with CD4^+ ^and CD8^+ ^T cell proliferation in both). CD4^+ ^T cell responses were of a similar magnitude to CD8^+ ^T cell responses (data not shown). No responses were detected in PBMC from 9 unexposed control blood donors.

As *Plasmodium *spp have an additional eight amino acids compared to human HG(X)PRT at the N-terminus, peptides #1 and #2 covering the first 30 amino acids from the N-terminal were evaluated in proliferation assays (Figure 3). PBMC from six selected acute malaria subjects known to respond to HGXPRT were evaluated and three responded to the peptides with CD4^+ ^and CD8^+ ^T cell proliferation, demonstrating T cell recognition of the N-terminus in a subset of individuals.

### Cytokine production in response to HGXPRT

**Figure 4:**
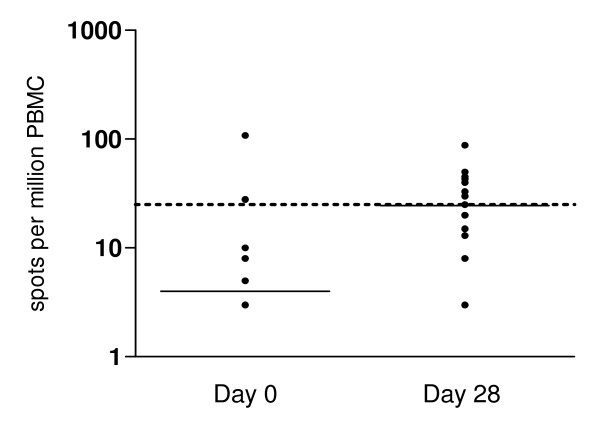
**Lymphocyte IFN-γ secretion to HGXPRT in acute and convalescent malaria: Ex-vivo ELISPOT detection of IFN-γ secretion following HGXPRT stimulation**. PBMC responses in 12 acute (day 0) and 19 convalescent (day 28) subjects are shown after background subtraction. Spot forming cells were significantly more numerous during convalescence (p = 0.04). The horizontal solid line represents the group median and the dotted line the background cut-off for positive responses.

IFN-γ has been identified as a cytokine capable of mediating potent anti-malarial immunity [30]. Therefore, PBMC collected during acute infection and in convalescence were tested for IFN-γ production in response to recombinant HGXPRT using ELISPOT. In contrast to the early detection of T cell proliferative responses, IFN-γ secretion was greater in convalescence (53%, 10/19 day 28) than during acute infection (17%, 2/12 day 0; p = 0.065), with significantly more numerous spot-forming cells (p = 0.04, Figure 4). All PBMC produced IFN-γ in response to PHA and no HGXPRT responses were detected in unexposed control samples. There were no significant differences in age, sex, baseline parasitaemia or response to treatment between the HGXPRT ELISPOT responders and non-responders (Table 2).

Among the 12 samples with sufficient cells to permit the evaluation of both HGXPRT proliferation and IFN-γ secretion, convalescent day 28 samples from six subjects responded in the ELISPOT assay but did not proliferate to HGXPRT. Two additional convalescent samples responded in neither assay. For the acute samples; three did not respond in the ELISPOT or proliferation assay while only proliferation was detected in the fourth sample. These results indicate dissociation between T cell proliferation and IFN-γ secretion in the T cell response to HGXPRT.

To examine whether the loss of proliferative responses in convalescence may be associated with Th2 cytokine production, culture supernatants from acute and convalescent PBMC (n = 4) following HGXPRT stimulation were also tested for IL-2, IL-4, IL-6, IL-10 and TNF cytokines. IL-6 (range 0.85–11.9 ng/mL), IL-10 (range 0.005–0.08 ng/mL), and TNF (range 0.03–4.4 ng/mL) were detected in supernatants from all acute and convalescent PBMC at each time-point (18, 36 and 65 hours) following HGXPRT stimulation. However, as noted for IFN-γ only IL-10 secretion increased in convalescence (day 7 or day 28) relative to paired acute infection samples, by a median of 35% (IQR 32–55%; p = 0.005).

### No detection of *Plasmodium *HGXPRT-specific IgG

**Figure 5:**
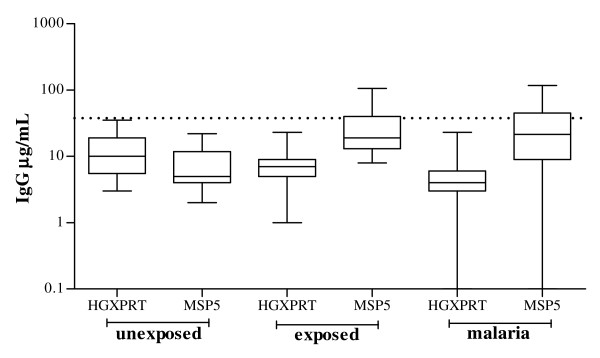
**Antibody responses to *P. falciparum *HGXPRT and MSP5**. Plasma IgG responses in 37 unexposed donors, 85 malaria exposed asymptomatic controls and 80 people with acute malaria (72 tested for MSP5 recognition). The solid line represents the group median and the dotted line the cut-off for positive responses as defined in the methods.

HGXPRT reactive immunoglobulin was assessed in the plasma of 80 Timika residents with acute malaria and 34 convalescent samples. The set included all people in whom cellular responses were tested plus an additional 85 asymptomatic Timika residents exposed to malaria for a minimum of two years. HGXPRT-specific IgG responses were not detected during acute infection nor 28 days after treatment in any subject, including when plasma was tested at a 1:400 dilution. The lack of HGXPRT-specific IgG responses contrasted with the frequent detection of convalescent IgG antibody responses to MSP5 in the same samples [27] (Figure 5).

## Discussion

This study represents the first characterisation of human cellular responses to *Plasmodium *HGXPRT, a blood stage antigen demonstrated to be recognized by protective T cells in pre-clinical studies [18]. Cellular recognition of *Plasmodium *HGXPRT during acute and convalescent malaria was identified, with antigen-specific proliferation frequently detected during acute infection but rarely in convalescence, and IFN-γ secretion detected predominantly during convalescence. These data show that HGXPRT is immunogenic in humans and suggest natural acute exposure frequently results in expansion of HGXPRT-specific T cells, which secrete IFN-γ in convalescence.

T cell proliferative responses to protein and peptides derived from *Plasmodium *HGXPRT were mediated by both CD4^+ ^and CD8^+ ^T cells, and CD8^+ ^T cell proliferation was CD4^+ ^T cell-dependent in accord with data from human studies with other antigens [31,32]. The frequent detection of HGXPRT proliferation only during acute infection is in agreement with longitudinal studies reporting short-lived proliferative responses following acute infection [33,34], but contrasts with other studies showing diminished proliferative responses during acute malaria infection and heightened responses in convalescence [35-37]. These differences may be due to the properties of the antigens tested and the genetic background and immune status of the population evaluated. In the current study, HGXPRT T cell proliferative responses diminished as the parasite burden was cleared in parallel with the increased detection of IFN-γ and IL-10 secretory responses. The loss of detectable HGXPRT-specific proliferative responses in convalescence may reflect the maturation of T cell responses from an acute primarily proliferative phase to an effector phase with elevated IL-10 [38], alternatively, the parasite-specific CD4^+ ^T cell responses may be deleted as occurs following rodent *Plasmodium *infection [39].

The more prevalent detection of HGXPRT-specific IFN-γ responses in convalescence was in accordance with other studies [36,40]. IFN-γ effector function appeared independent of the parasite burden and independent of proliferation. The dissociation between proliferative and secretory responses is in agreement with studies of HGXPRT T cell responses in a murine model of natural immunity (Yawalak Panpisutchai, personal communication) and human responses to other malaria antigens [41-43].

Limitations of this study include an inability to study HLA- and ethnically-matched malaria unexposed controls, however, the use of Australian controls showed that proliferative responses in the malaria-exposed individuals were not non-specific or a mitogen response. Given the sequence conservation between *Homo sapiens *and *Plasmodium *HG(X)PRT, it is possible that the cellular responses identified in the present study may recognize structures close to self; also there is potential for HGXPRT vaccination to induce autoimmunity. While further development of this antigen would require exclusion of these possibilities, the characterisation of minimal epitopes conserved among *Plasmodium *species but distinct from human HG(X)PRT would mitigate against this potential.

No HGXPRT IgG reactivity was identified in the study cohort tested, suggesting natural exposure fails to stimulate memory B cell responses. The absence of HGXPRT IgG was not caused by lack of sufficient malaria exposure or a deficit in IgG production as all asymptomatic subjects had lived in a malaria-endemic area for a period of at least two years, and plasma MSP5 IgG responses were frequently detected in the same patients [27]. The absence of IgG reactivity distinguishes HGXPRT from the majority of other malaria antigens.

The detection of both CD4^+ ^and CD8^+ ^HGXPRT reactive T cells and the absence of HGXPRT specific IgG responses in people with acute malaria indicates that natural exposure generates different immune responsiveness to that of the *P. yoelii *experimental murine malaria model. Such differences illustrate the importance of evaluating immunological responses in human infection.

## Conclusion

CD4 T cells recognizing HGXPRT confer protection in a murine malaria model and the current study now demonstrates robust T cell proliferation to *Plasmodium *HGXPRT protein and peptides during human malaria infection. Further studies to determine minimal HGXPRT epitopes, the specificity of responses for *Plasmodium *spp. and associations with protection are required. Frequent and robust T cell recognition, high sequence conservation among *Plasmodium *spp. and absent IgG responses distinguish HGXPRT from other malaria antigens.

## Competing interests

The authors declare that they have no competing interests.

## Authors' contributions

Study conceptualization and design: TW, APC, CRE, DLD, MG, NMA. Cohort recruitment and sample processing: KP, RNP, EK, ET, NMA. HGXPRT recombinant protein preparation: YP. Testing of PBMC & plasma: TW, KP, ES. Data analysis: TW, KP. Manuscript preparation: TW, APC, KP, YP, CRE, ES, EK, ET, RNP, DLD, MG, NMA. All authors have read and approved the final manuscript.

*Conference presentations*:

Keystone Symposia, Malaria: Immunology, Pathogenesis and Vaccine Perspectives June 2008 Alpbach, Austria. Oral presentation. Human T cell recognition of the novel vaccine target *Plasmodium *HGXPRT following natural parasite exposure.

## References

[B1] Snow RW, Guerra CA, Noor AM, Myint HY, Hay SI (2005). The global distribution of clinical episodes of *Plasmodium falciparum *malaria. Nature.

[B2] Clyde DF, Most H, McCarthy VC, Vanderberg JP (1973). Immunization of man against sporozite-induced falciparum malaria. Am J Med Sci.

[B3] Hoffman SL, Goh LM, Luke TC, Schneider I, Le TP, Doolan DL, Sacci J, de la Vega P, Dowler M, Paul C (2002). Protection of humans against malaria by immunization with radiation-attenuated *Plasmodium falciparum *sporozoites. J Infect Dis.

[B4] Pombo DJ, Lawrence G, Hirunpetcharat C, Rzepczyk C, Bryden M, Cloonan N, Anderson K, Mahakunkijcharoen Y, Martin LB, Wilson D (2002). Immunity to malaria after administration of ultra-low doses of red cells infected with *Plasmodium falciparum*. Lancet.

[B5] Edstein MD, Kotecka BM, Anderson KL, Pombo DJ, Kyle DE, Rieckmann KH, Good MF (2005). Lengthy antimalarial activity of atovaquone in human plasma following atovaquone-proguanil administration. Antimicrob Agents Chemother.

[B6] Bejon P, Lusingu J, Olotu A, Leach A, Lievens M, Vekemans J, Mshamu S, Lang T, Gould J, Dubois MC (2008). Efficacy of RTS,S/AS01E vaccine against malaria in children 5 to 17 months of age. N Engl J Med.

[B7] Abdulla S, Oberholzer R, Juma O, Kubhoja S, Machera F, Membi C, Omari S, Urassa A, Mshinda H, Jumanne A (2008). Safety and immunogenicity of RTS,S/AS02D malaria vaccine in infants. N Engl J Med.

[B8] Alonso PL, Sacarlal J, Aponte JJ, Leach A, Macete E, Aide P, Sigauque B, Milman J, Mandomando I, Bassat Q (2005). Duration of protection with RTS,S/AS02A malaria vaccine in prevention of *Plasmodium falciparum *disease in Mozambican children: single-blind extended follow-up of a randomised controlled trial. Lancet.

[B9] Dunachie SJ, Walther M, Epstein JE, Keating S, Berthoud T, Andrews L, Andersen RF, Bejon P, Goonetilleke N, Poulton I (2006). A DNA prime-modified vaccinia virus ankara boost vaccine encoding thrombospondin-related adhesion protein but not circumsporozoite protein partially protects healthy malaria-naive adults against *Plasmodium falciparum *sporozoite challenge. Infect Immun.

[B10] Nussenzweig V, Nussenzweig RS (1989). Rationale for the development of an engineered sporozoite malaria vaccine. Adv Immunol.

[B11] Doolan DL, Martinez-Alier N (2006). Immune response to pre-erythrocytic stages of malaria parasites. Curr Mol Med.

[B12] Grun JL, Weidanz WP (1981). Immunity to *Plasmodium chabaudi adami *in the B-cell-deficient mouse. Nature.

[B13] Cavacini LA, Parke LA, Weidanz WP (1990). Resolution of acute malarial infections by T cell-dependent non-antibody-mediated mechanisms of immunity. Infect Immun.

[B14] Spitalny GL, Verhave JP, Meuwissen JH, Nussenzweig RS (1977). *Plasmodium berghei*: T cell dependence of sporozoite-induced immunity in rodents. Exp Parasitol.

[B15] Egan JE, Weber JL, Ballou WR, Hollingdale MR, Majarian WR, Gordon DM, Maloy WL, Hoffman SL, Wirtz RA, Schneider I (1987). Efficacy of murine malaria sporozoite vaccines: implications for human vaccine development. Science.

[B16] Weiss WR, Sedegah M, Beaudoin RL, Miller LH, Good MF (1988). CD8+ T cells (cytotoxic/suppressors) are required for protection in mice immunized with malaria sporozoites. Proc Natl Acad Sci USA.

[B17] Tsuji M, Romero P, Nussenzweig RS, Zavala F (1990). CD4+ cytolytic T cell clone confers protection against murine malaria. J Exp Med.

[B18] Makobongo MO, Riding G, Xu H, Hirunpetcharat C, Keough D, de Jersey J, Willadsen P, Good MF (2003). The purine salvage enzyme hypoxanthine guanine xanthine phosphoribosyl transferase is a major target antigen for cell-mediated immunity to malaria. Proc Natl Acad Sci USA.

[B19] Shahabuddin M, Gunther K, Lingelbach K, Aikawa M, Schreiber M, Ridley RG, Scaife JG (1992). Localisation of hypoxanthine phosphoribosyl transferase in the malaria parasite *Plasmodium falciparum*. Exp Parasitol.

[B20] Reyes P, Rathod PK, Sanchez DJ, Mrema JE, Rieckmann KH, Heidrich HG (1982). Enzymes of purine and pyrimidine metabolism from the human malaria parasite, *Plasmodium falciparum*. Mol Biochem Parasitol.

[B21] Karyana M, Burdarm L, Yeung S, Kenangalem E, Wariker N, Maristela R, Umana KG, Vemuri R, Okoseray MJ, Penttinen PM (2008). Malaria morbidity in Papua Indonesia, an area with multidrug resistant *Plasmodium vivax *and *Plasmodium falciparum*. Malar J.

[B22] Ratcliff A, Siswantoro H, Kenangalem E, Maristela R, Wuwung RM, Laihad F, Ebsworth EP, Anstey NM, Tjitra E, Price RN (2007). Two fixed-dose artemisinin combinations for drug-resistant falciparum and vivax malaria in Papua, Indonesia: an open-label randomised comparison. Lancet.

[B23] Ratcliff A, Siswantoro H, Kenangalem E, Wuwung M, Brockman A, Edstein MD, Laihad F, Ebsworth EP, Anstey NM, Tjitra E (2007). Therapeutic response of multidrug-resistant *Plasmodium falciparum *and *P. vivax *to chloroquine and sulfadoxine-pyrimethamine in southern Papua, Indonesia. Trans R Soc Trop Med Hyg.

[B24] Bahl A, Brunk B, Crabtree J, Fraunholz MJ, Gajria B, Grant GR, Ginsburg H, Gupta D, Kissinger JC, Labo P (2003). PlasmoDB: the Plasmodium genome resource. A database integrating experimental and computational data. Nucleic Acids Res.

[B25] Keough DT, Ng AL, Winzor DJ, Emmerson BT, de Jersey J (1999). Purification and characterization of Plasmodium falciparum hypoxanthine-guanine-xanthine phosphoribosyltransferase and comparison with the human enzyme. Mol Biochem Parasitol.

[B26] King A, Melton DW (1987). Characterisation of cDNA clones for hypoxanthine-guanine phosphoribosyltransferase from the human malarial parasite, Plasmodium falciparum: comparisons to the mammalian gene and protein. Nucleic Acids Res.

[B27] Woodberry T, Minigo G, Piera KA, Hanley JC, de Silva HD, Salwati E, Kenangalem E, Tjitra E, Coppel RL, Price RN (2008). Antibodies to Plasmodium falciparum and Plasmodium vivax merozoite surface protein 5 in Indonesia: species-specific and cross-reactive responses. J Infect Dis.

[B28] Flanagan KL, Lee EA, Gravenor MB, Reece WH, Urban BC, Doherty T, Bojang KA, Pinder M, Hill AV, Plebanski M (2001). Unique T cell effector functions elicited by Plasmodium falciparum epitopes in malaria-exposed Africans tested by three T cell assays. J Immunol.

[B29] Minigo G, Woodberry T, Piera KA, Salwati E, Tjitra E, Kenangalem E, Price RN, Engwerda CR, Anstey NM, Plebanski M (2009). Parasite-dependent expansion of TNF receptor II-positive regulatory T cells with enhanced suppressive activity in adults with severe malaria. PLoS Pathog.

[B30] Angulo I, Fresno M (2002). Cytokines in the pathogenesis of and protection against malaria. Clin Diagn Lab Immunol.

[B31] Wang R, Epstein J, Baraceros FM, Gorak EJ, Charoenvit Y, Carucci DJ, Hedstrom RC, Rahardjo N, Gay T, Hobart P (2001). Induction of CD4(+) T cell-dependent CD8(+) type 1 responses in humans by a malaria DNA vaccine. Proc Natl Acad Sci USA.

[B32] Wang R, Epstein J, Charoenvit Y, Baraceros FM, Rahardjo N, Gay T, Banania JG, Chattopadhyay R, de la Vega P, Richie TL (2004). Induction in humans of CD8+ and CD4+ T cell and antibody responses by sequential immunization with malaria DNA and recombinant protein. J Immunol.

[B33] Troye-Blomberg M, Romero P, Patarroyo ME, Bjorkman A, Perlmann P (1984). Regulation of the immune response in Plasmodium falciparum malaria. III. Proliferative response to antigen in vitro and subset composition of T cells from patients with acute infection or from immune donors. Clin Exp Immunol.

[B34] de Oliveira-Ferreira J, Banic DM, Santos F, Ferreira-da-Cruz MF, Dubois P, Daniel-Ribeiro CT (1999). Cellular and antibody responses to the *Plasmodium falciparum *heat shock protein Pf72/HSP70 during and after acute malaria in individuals from an endemic area of Brazil. Acta Trop.

[B35] Ho M, Webster HK, Looareesuwan S, Supanaranond W, Phillips RE, Chanthavanich P, Warrell DA (1986). Antigen-specific immunosuppression in human malaria due to *Plasmodium falciparum*. J Infect Dis.

[B36] Riley EM, Andersson G, Otoo LN, Jepsen S, Greenwood BM (1988). Cellular immune responses to *Plasmodium falciparum *antigens in Gambian children during and after an acute attack of falciparum malaria. Clin Exp Immunol.

[B37] Theander TG, Bygbjerg IC, Andersen BJ, Jepsen S, Kharazmi A, Odum N (1986). Suppression of parasite-specific response in *Plasmodium falciparum *malaria. A longitudinal study of blood mononuclear cell proliferation and subset composition. Scand J Immunol.

[B38] Joss A, Akdis M, Faith A, Blaser K, Akdis CA (2000). IL-10 directly acts on T cells by specifically altering the CD28 co-stimulation pathway. Eur J Immunol.

[B39] Xu H, Wipasa J, Yan H, Zeng M, Makobongo MO, Finkelman FD, Kelso A, Good MF (2002). The mechanism and significance of deletion of parasite-specific CD4(+) T cells in malaria infection. J Exp Med.

[B40] Bejon P, Mwacharo J, Kai O, Todryk S, Keating S, Lowe B, Lang T, Mwangi TW, Gilbert SC, Peshu N (2007). The induction and persistence of T cell IFN-gamma responses after vaccination or natural exposure is suppressed by *Plasmodium falciparum*. J Immunol.

[B41] Good MF, Pombo D, Quakyi IA, Riley EM, Houghten RA, Menon A, Alling DW, Berzofsky JA, Miller LH (1988). Human T-cell recognition of the circumsporozoite protein of *Plasmodium falciparum*: immunodominant T-cell domains map to the polymorphic regions of the molecule. Proc Natl Acad Sci USA.

[B42] Connelly M, King CL, Bucci K, Walters S, Genton B, Alpers MP, Hollingdale M, Kazura JW (1997). T-cell immunity to peptide epitopes of liver-stage antigen 1 in an area of Papua New Guinea in which malaria is holoendemic. Infect Immun.

[B43] Joshi SK, Bharadwaj A, Chatterjee S, Chauhan VS (2000). Analysis of immune responses against T- and B-cell epitopes from *Plasmodium falciparum *liver-stage antigen 1 in rodent malaria models and malaria-exposed human subjects in India. Infect Immun.

